# Cerebromicrovascular mechanisms contributing to long COVID: implications for neurocognitive health

**DOI:** 10.1007/s11357-024-01487-4

**Published:** 2025-01-07

**Authors:** Monika Fekete, Andrea Lehoczki, Ágnes Szappanos, Attila Toth, Mohamed Mahdi, Péter Sótonyi, Zoltán Benyó, Andriy Yabluchanskiy, Stefano Tarantini, Zoltan Ungvari

**Affiliations:** 1 Institute of Preventive Medicine and Public Health, Semmelweis University, Budapest, Hungary; 2 Doctoral College, Health Sciences Program, Semmelweis University, Budapest, Hungary; 3 Heart and Vascular Center, Semmelweis University, Budapest, Hungary; 4 Department of Rheumatology and Clinical Immunology, Semmelweis University, Budapest, Hungary; 5 Division of Clinical Physiology, Department of Cardiology, Faculty of Medicine, University of Debrecen, Debrecen, 4032 Hungary; 6 Research Centre for Molecular Medicine, University of Debrecen, Debrecen, 4032 Hungary; 7 Laboratory of Retroviral Biochemistry, Department of Biochemistry and Molecular Biology, University of Debrecen, 4032 Debrecen, Hungary; 8 Infectology Clinic, University of Debrecen Clinical Centre, 4031 Debrecen, Hungary; 9 Department of Vascular and Endovascular Surgery, Heart and Vascular Centre, Semmelweis University, 1122 Budapest, Hungary; 10 Institute of Translational Medicine, Semmelweis University, 1094 Budapest, Hungary; 11 Cerebrovascular and Neurocognitive Disorders Research Group, HUN-REN (魂人), Semmelweis University, 1094 Budapest, Hungary; 12 Vascular Cognitive Impairment, Neurodegeneration and Healthy Brain Aging Program, Department of Neurosurgery, University of Oklahoma Health Sciences Center, Oklahoma City, OK USA; 13 International Training Program in Geroscience, Doctoral College/Institute of Preventive Medicine and Public Health, Semmelweis University, Budapest, Hungary; 14 Stephenson Cancer Center, University of Oklahoma, Oklahoma City, OK USA; 15 Oklahoma Center for Geroscience and Healthy Brain Aging, University of Oklahoma Health Sciences Center, Oklahoma City, OK USA; 16 Department of Health Promotion Sciences, College of Public Health, University of Oklahoma Health Sciences Center, Oklahoma City, OK USA

**Keywords:** Long COVID, Cerebromicrovascular dysfunction, Endothelial dysfunction, Mitochondrial dysfunction, Neurovascular coupling, Autoantibodies, Long-haul COVID, Persistent COVID symptoms

## Abstract

Long COVID (also known as post-acute sequelae of SARS-CoV-2 infection [PASC] or post-COVID syndrome) is characterized by persistent symptoms that extend beyond the acute phase of SARS-CoV-2 infection, affecting approximately 10% to over 30% of those infected. It presents a significant clinical challenge, notably due to pronounced neurocognitive symptoms such as brain fog. The mechanisms underlying these effects are multifactorial, with mounting evidence pointing to a central role of cerebromicrovascular dysfunction. This review investigates key pathophysiological mechanisms contributing to cerebrovascular dysfunction in long COVID and their impacts on brain health. We discuss how endothelial tropism of SARS-CoV-2 and direct vascular infection trigger endothelial dysfunction, impaired neurovascular coupling, and blood–brain barrier disruption, resulting in compromised cerebral perfusion. Furthermore, the infection appears to induce mitochondrial dysfunction, enhancing oxidative stress and inflammation within cerebral endothelial cells. Autoantibody formation following infection also potentially exacerbates neurovascular injury, contributing to chronic vascular inflammation and ongoing blood–brain barrier compromise. These factors collectively contribute to the emergence of white matter hyperintensities, promote amyloid pathology, and may accelerate neurodegenerative processes, including Alzheimer’s disease. This review also emphasizes the critical role of advanced imaging techniques in assessing cerebromicrovascular health and the need for targeted interventions to address these cerebrovascular complications. A deeper understanding of the cerebrovascular mechanisms of long COVID is essential to advance targeted treatments and mitigate its long-term neurocognitive consequences.

## Introduction

Long COVID, also known as post-acute sequelae of SARS-CoV-2 infection (PASC), has emerged as a major healthcare challenge in the wake of the COVID-19 pandemic [1–10]. While the acute phase of COVID-19 predominantly affects the respiratory system, viremia—although less common in mild and moderate cases—has been observed in severe cases, facilitating the systemic spread of the virus to various tissues [11]. Emerging evidence indicates that COVID-19 can exert profound and lasting effects on multiple organ systems, including the central nervous system (CNS) [12–15]. Long COVID encompasses a spectrum of persistent neurocognitive symptoms and manifestations that persist for weeks or months after the acute phase of SARS-CoV-2 infection [16]. Common symptoms reported by individuals with long COVID include persistent fatigue, cognitive dysfunction (often referred to as “brain fog”), anosmia, ageusia, sleep disturbances, and mood disorders such as depression and anxiety [16, 17]. Additionally, neurological symptoms such as headache and dizziness have been frequently reported [17]. Some individuals may also experience post-exertional malaise, where even mild physical or cognitive exertion results in extreme fatigue and exacerbation of symptoms. These ongoing symptoms can severely impact quality of life, limiting individuals’ ability to perform daily activities and diminishing work productivity. Long COVID poses a particularly significant challenge for working-age adults, who may experience a disproportionate burden due to their inability to return to work or resume daily activities. This midlife stressor not only impacts physical and mental health but also has economic and social consequences, further magnified in working-age populations [18].

Estimating the true incidence and prevalence of long COVID presents significant challenges due to variations in definitions, study methodologies, and the evolving nature of the condition [1–7]. Community studies have highlighted the varied prevalence and societal impact of long COVID across different populations. For example, regional disparities in long COVID outcomes have been documented [19, 20]. Early reports suggested that a substantial proportion of individuals with COVID-19 experienced persistent symptoms beyond the acute phase of infection, with estimates ranging from 10 to over 30% of individuals infected with SARS-CoV-2 [1–7]. A recent meta-analysis of 196 studies, consisting of 120,970 participants indicates that the prevalence of long COVID may be higher (over 50%), particularly among those with severe or prolonged illness [8]. Long COVID affects individuals across all age groups, including those with mild or asymptomatic/paucisymptomatic initial infections, although older adults and individuals with pre-existing comorbidities are at higher risk [1–7]. Neurocognitive long COVID symptomatology is most frequent in older adults [8]. The persisting neurocognitive effects of COVID-19 in older adults are of significant concern, particularly given their heightened vulnerability to severe illness and increased risk for developing dementia [21–23].

Among the neurological sequelae of COVID-19, cerebromicrovascular causes have garnered increasing attention due to their potential implications for long-term brain health and cognitive function in older adults [21, 24, 25]. This review examines the role of the cerebromicrovascular system in the pathogenesis of long COVID, focusing on its impact on brain health, cognitive function, and the development of neurological sequelae. By synthesizing current evidence and identifying gaps in knowledge, this review aims to provide insights into potential therapeutic strategies and directions for future research in this emerging field.

## Importance of understanding cerebromicrovascular contributions to long COVID

Mounting evidence indicates that microvascular dysfunction and injury play a pivotal role in mediating COVID-19-related injury to the heart [26, 27], kidney [28–30], lungs [31, 32], and other organs [33]. There is also growing evidence that cerebromicrovascular injury and dysfunction also contribute to SARS-CoV-2 infection-related brain injury and the perpetuation of neurological symptoms among long COVID patients [21, 25, 34–43]. Here we discuss possible mechanisms underpinning this association, as well as potential therapeutic avenues that hold promise for enhancing the quality of life of those grappling with these enduring symptoms.

### Role of cerebromicrovascular health in maintaining brain health

The preservation of normal brain function relies heavily on the maintenance of adequate tissue perfusion through a dense cerebromicrovascular network [44–48]. Remarkably, the total length of capillaries in the human brain spans approximately 600 km, with virtually every neuron being supplied by its own capillary. As individuals age, growing evidence indicates the occurrence of multifaceted functional impairment within the cerebral microcirculation, significantly contributing to brain aging and the onset of age-related cognitive impairment [49–55]. In an effort to recognize the contribution of cerebrovascular mechanisms to cognitive decline, the term “vascular cognitive impairment and dementia (VCID)” was coined. The concept of VCID underscores the idea that a spectrum of vascular pathologies, including dysregulation of cerebral blood flow (CBF), stroke, microinfarcts, microhemorrhages/microbleeds, blood–brain barrier (BBB) disruption and consequential neuroinflammation and leukoaraiosis, and cerebral amyloid angiopathy, can collectively promote cognitive impairment in older individuals. For the purposes of this review, our focus is specifically on the role of cerebrovascular pathologies related to SARS-CoV-2 in the development of chronic neurological manifestations associated with long COVID.

The regulation of CBF must adhere to unique requirements to ensure the consistent delivery of vital nutrients and oxygen to the brain while avoiding both hypoperfusion and hyperperfusion. Several factors contribute to this complexity: first, the brain’s remarkably high metabolic demand for oxygen relative to other organs; second, neurons’ limited energy reserves; third, the rapid fluctuations in metabolic demand with neuronal activation; fourth, the constraint of space within the closed cranium, necessitating the maintenance of normal blood flow, volume, and intracranial pressure; and fifth, the prevention of high-pressure penetration into the distal and vulnerable segments of the cerebral arterial tree, which could lead to microvascular damage and BBB disruption [56]. To meet these demanding requirements, the regulation of CBF relies on an intricate network of overlapping regulatory mechanisms. Mounting evidence suggests that even mild impairments in microvascular function and CBF regulation can have significant consequences for cerebral function. Within this complex regulatory framework, the health of the endothelium and the integrity of the BBB assume critical roles in preserving brain health. The microvascular endothelium plays a key role in CBF regulation, maintenance of capillary network architecture, and the BBB. It also regulates hemostasis. Simultaneously, the microvascular endothelium within the neurogenic niche plays a pivotal role by secreting essential growth factors that influence neurogenesis. The BBB controls the passage of substances into the brain, thereby shielding it from potentially harmful agents. In the context of this review, we explore the impact of COVID-19 on the cerebral circulation, focusing on potential mechanisms underlying cerebrovascular dysfunction and its subsequent pathophysiological consequences related to long COVID.

### Vulnerability of the microcirculation to COVID-19 infection

A critical aspect of understanding the cerebromicrovascular causes in the context of long COVID is the susceptibility of cerebromicrovascular endothelial cells to infection by the SARS-CoV-2 virus. Endothelial cells, which form the inner lining of blood vessels, have garnered significant attention due to their pivotal role in vascular function and their interactions with viruses. Of particular interest is the substantial expression of angiotensin-converting enzyme-2 (ACE-2) on endothelial cells, which serves as the primary entry receptor for SARS-CoV-2 [57]. Several notable studies, employing diverse detection techniques, including electron microscopy, have convincingly demonstrated the presence of SARS-CoV-2 within microvascular endothelial cells [58–61]. Consequently, SARS-CoV-2 is now acknowledged as an endotheliotrophic virus, akin to cytomegalovirus and certain herpes viruses [62–64]. These viruses establish latent infections with periodic reactivation, leading to long-term endothelial dysfunction. Notably, this persistent endothelial damage has been implicated in post-infection syndromes that share similarities with long COVID, as highlighted in recent studies [65–72]. This shared pathogenic pathway emphasizes the critical role of endothelial health in the development of post-viral syndromes and highlights its broader implications for understanding and managing long-term sequelae [67–72].

The infection and inflammation induced by SARS-CoV-2 within the microvasculature are regarded as pivotal components of the pathogenesis of COVID-19 [73–80]. The sequence of events begins with the spike protein of SARS-CoV-2 binding to ACE2 receptors on endothelial cells, facilitating viral entry and infection. This process initiates a complex cascade of events. The virus hijacks the cellular machinery for replication, significantly increasing metabolic demands and leading to structural and functional disruption of the infected cells. These cytopathic effects may result in endothelial cell death and contribute to widespread vascular damage. Consequently, profound functional and phenotypic changes occur within cerebromicrovascular endothelial cells [81–84]. Viral entry leads to downregulation and internalization of ACE2, disrupting the delicate balance of the renin-angiotensin system (RAS) [85]. Specifically, ACE2 downregulation decreases the conversion of angiotensin II into angiotensin 1–7, resulting in elevated levels of angiotensin II and reduced levels of angiotensin 1–7. The imbalance favors vasoconstriction, inflammation, and endothelial dysfunction, as angiotensin II promotes pro-inflammatory cytokine release and oxidative stress, while the loss of angiotensin 1–7 reduces protective, anti-inflammatory signaling. This RAS dysregulation amplifies vascular damage and may contribute to the chronic endothelial injury observed in long COVID. The culmination of these changes gives rise to the development of a thromboinflammatory environment characterized by the excessive production of reactive oxygen species (ROS) [86], altered barrier function, and physical damage to capillaries [87, 88]. These multifaceted alterations not only induce endothelial vasomotor dysfunction but also disrupt the delicate equilibrium of endothelial function within the macro- and microvasculature. Importantly, these effects manifest in both acute COVID-19 cases and individuals grappling with the enduring symptoms of long COVID [34, 41, 42, 89, 90]. Additionally, endotheliitis and microvascular thrombosis might also be triggered by indirect effects of SARS-CoV-2 infection [91].

## Impact of COVID-19 on cerebral microvasculature and its implications for long COVID

A growing body of research has been dedicated to understanding the long-lasting impact of COVID-19 on the cerebromicrovascular system [12, 13, 92–97]. The consequences of endotheliitis and SARS-CoV-2-induced cerebromicrovascular dysfunction have far-reaching implications that likely encompass a spectrum of effects, including impaired CBF, disruption of the BBB, and perivascular inflammation. This sequence of interconnected events can lead to a multitude of adverse outcomes, including reduced oxygen supply to brain cells, heightened thrombogenesis, the release of pro-inflammatory factors, the onset of neuroinflammation, white matter damage, and disruption of the structural integrity of cerebral microvessels [24, 25]. Each of these factors is likely to contribute significantly to the enduring neurological manifestations associated with long COVID. Over time, the cumulative impact of these factors can culminate in cognitive impairment, an increased susceptibility to neurodegenerative diseases, and heightened vulnerability to various neurological disorders. Consequently, the understanding and comprehensive addressing of cerebromicrovascular causes assume a pivotal role in mitigating the long-term brain complications associated with long COVID.

### Age-related susceptibility, microvascular aging, and neurological impact of long COVID

COVID-19 demonstrates a marked age-related severity gradient, consistently documented in an extensive body of literature that emphasizes the heightened risk and increased disease severity observed in older adults [98–103]. Within this demographic, there is a notably elevated incidence of acute and post-COVID-19 complications, prominently featuring chronic neurological and vascular issues [104–108]. While major vascular events are typically not categorized as part of long COVID, it is essential to acknowledge that COVID-19 can still induce considerable morbidity and mortality from such events [109–117], which may be underestimated or underreported [118–122]. COVID-19 has been observed to promote thromboembolism, including the formation of microthrombi [123, 124]. It is conceivable that undiagnosed small strokes could contribute to symptoms diagnosed as long COVID. Small strokes, also known as “silent strokes,” can occur without noticeable symptoms or may cause subtle symptoms that are easily overlooked or attributed to other factors. Importantly, during the COVID-19 pandemic, there was a decline in stroke and transient ischemic attack admissions, potentially due to hesitancy in seeking medical attention [118, 121, 125]. “Silent” strokes can affect cognitive function, mood, and overall well-being, which are also symptoms commonly associated with long COVID. Therefore, it is plausible that undiagnosed small strokes may contribute to the constellation of symptoms experienced by individuals diagnosed with long COVID. However, further research is needed to better understand the potential relationship between undiagnosed small strokes and long COVID symptoms. The epidemiological data raise alarms regarding the long-term cardiovascular and cerebrovascular consequences of COVID-19 [120]. Therefore, understanding the full spectrum of cardiovascular and cerebrovascular implications, including age-related trends, is crucial for assessing the comprehensive impact of COVID-19 beyond acute infection.

Age appears to play a crucial role in shaping the risk and severity of long COVID [126–128]. Older individuals have a heightened likelihood of experiencing persistent symptoms and complications following acute SARS-CoV-2 infection [129]. A recent study, part of the RECOVER initiative funded by the United States National Institutes of Health, examined the relationship between SARS-CoV-2 vaccination and long COVID diagnosis using electronic health records from the National COVID Cohort Collaborative [130]. Results showed that vaccination was consistently associated with lower odds and rates of long COVID diagnosis [130]. Additionally, older adults, despite being more likely to be vaccinated, were also more prone to contracting long COVID compared to younger adults [130]. Advancing age also emerges as a risk factor for the development of post-COVID neurocognitive manifestations [126, 128]. This age-related vulnerability may stem from various factors [131], including age-related declines in immune function, pre-existing health conditions, and the cumulative effects of accelerated physiological aging on organ systems. Additionally, older adults exhibit heightened systemic inflammation and often have a higher prevalence of comorbidities, such as hypertension, diabetes mellitus, cardiovascular disease, and pathologies affecting the hematopoietic system (including clonal hematopoiesis of indeterminate potential [CHIP] [132–138] and monoclonal gammopathy of undetermined significance [MGUS] [138]), which can exacerbate the impact of COVID-19 and contribute to the development of long-lasting symptoms [1]. For similar reasons, older adults are also prone to experiencing long-term cognitive consequences associated with hospitalization for other diseases [139]. It is crucial to recognize that socioeconomic, genetic, and epigenetic factors associated with aging may also play a significant role in susceptibility to infection and long COVID outcomes. Epigenetic modifications have been identified as critical regulators of immune function and aging, influencing immune cell activity and shaping individual vulnerability to infections and autoimmune disorders. By modulating gene expression, these modifications impact immune responses, ultimately determining the clinical spectrum and outcomes of both acute and chronic infections [140, 141]).

Conceptually, the heightened vulnerability of the aging microcirculation to stressors associated COVID-19 with can be attributed to several key factors. Firstly, aging is intricately linked with impaired endothelial function, which manifests as reduced vasodilation and an elevated state of oxidative stress [142–148] and inflammation within the vasculature [149–156]. Consequently, in older adults, cerebromicrovascular impairment induced by COVID-19 is compounded by pre-existing deficits in the functionality of the aging microcirculation. Secondly, microvascular rarefaction, marked by a decline in capillary density [157–159], characterizes the aging process, limiting the microvasculature’s ability to efficiently supply oxygen and nutrients to the brain [160–163]. This likely exacerbates the adverse consequences of microvascular dysregulation and inflammation during the pathogenesis of COVID-19, creating regions at heightened risk for ischemic damage. Thirdly, aging may impair autoregulatory mechanisms within the cerebral circulation [164–166], diminishing their capacity to adapt blood flow in response to evolving hemodynamic conditions, which could potentially contribute to exacerbated neurological damage during the acute phase of the disease [56, 167–169]. Fourthly, the persistent low-grade inflammation associated with aging, often termed inflammaging [170–173], plays a pivotal role in endothelial dysfunction and microvascular damage [149, 150, 174]. This pro-inflammatory environment, characterized by elevated levels of cytokines such as IL-6 and TNF-α, creates conditions favorable for viral replication by altering the local tissue milieu, thereby enhancing viral infectivity and propagation. Importantly, this state of chronic inflammation not only facilitates viral processes during the acute phase but may also contribute significantly to the development of long-term sequelae, including those observed in long COVID [127, 175]. The heightened state of systemic inflammation due to viral infection is likely to exacerbate cerebromicrovascular pathologies, including endothelial dysfunction and blood–brain barrier (BBB) disruption. This concept is reinforced by findings indicating the continued presence of pro-inflammatory factors in the serum of long COVID patients, which are capable of inducing endothelial activation [176]. Fifthly, aging associates with mitochondrial dysfunction [154, 155, 177–186], increased cellular production of reactive oxygen species, and impaired cellular resilience to oxidative stress, including the compromise of Nrf2-driven antioxidative response mechanisms [187–193]. The increased production of ROS and heightened oxidative stress within the aging microvasculature during COVID-19 can intensify damage to microvascular endothelial cells and disrupt their normal function. Sixthly, age-related comorbidities such as hypertension, diabetes [194–196], and cardiovascular disease further compound microvascular dysfunction [162, 168, 189, 197–206], with these comorbidities recognized as risk factors for severe COVID-19 and long COVID. Lastly, age-related alterations in the immune system (immunosenescence) may hinder its ability to mount an effective response against microvascular viral infections like SARS-CoV-2 [99, 140, 207–210], potentially facilitating greater viral dissemination and damage within the cerebral microcirculation. Collectively, these factors render the aging microcirculation notably more susceptible to damage induced by COVID-19. A comprehensive understanding of these age-related changes within the microvascular system is pivotal for comprehending the intricate neurological consequences associated with conditions like long COVID.

### Endothelial dysfunction, dysregulation of CBF and neurovascular uncoupling

Endothelial cells play a central and multifaceted role in the regulation of CBF, including mediation of neurovascular coupling (NVC) responses [56, 203, 211–214]. These functions are critical for maintaining proper brain function and ensuring that neurons receive the necessary oxygen and nutrients during various physiological activities. The endothelium actively participates in the regulation of CBF by producing and releasing various signaling molecules, such as nitric oxide (NO), prostacyclin, and endothelin. These mediators help to modulate the tone of the adjacent smooth muscle cells in the blood vessel walls, thereby influencing the diameter of cerebral resistance vessels and the amount of blood that flows through them. NVC is a fundamental homeostatic process that links neuronal activity with changes in CBF. When neurons become active and fire action potentials, they require increased oxygen and glucose. In response to this increased metabolic demand, nearby arterioles should dilate to deliver more blood to the active regions. Endothelial cells play a pivotal role in initiating this response [154–156, 215–217]. They sense the neural activity through various signaling astrocyte-dependent and astrocyte-independent pathways and, in turn, release vasodilatory molecules like NO. This local vasodilation ensures that the brain’s energy needs are met promptly and efficiently during cognitive tasks, sensory processing, or other neuronal activities. When NVC is impaired, the relationship between neuronal activity and blood flow becomes disrupted, resulting in inadequate delivery of oxygen and nutrients to active neurons. Additionally, the washout of toxic metabolic by-products is also compromised. This mismatch can lead to suboptimal neuronal function and, in severe cases, neuronal damage. Both the natural aging process and risk factors that accelerate cardiovascular aging, such as diabetes mellitus, hypertension, obesity, and smoking, can significantly compromise NVC responses through the promotion of endothelial dysfunction [162, 168, 189, 197–206].

In the context of COVID-19, emerging evidence indicates that SARS-CoV-2 infects endothelial cells. This viral infection together with the adverse endothelial effects of inflammatory mediators released from other organs lead to widespread endothelial dysfunction and potentially impair NVC responses [21, 24]. During the acute phase of COVID-19, it has been demonstrated that COVID-19-induced systemic macro- and microvascular endothelial dysfunction can exacerbate the severity of the disease [218–222]. The presence of viral antigens long after the acute infection can sustain inflammation and immune responses, further disrupting normal NVC function [223]. Although direct viral replication in the brain is uncommon in most cases, evidence suggests that SARS-CoV-2 can infect neuronal tissue, causing direct damage and alterations to NV [224, 225]. Additionally, immune responses directed at the virus, along with immune complex deposition, may impair NVC through vascular inflammation and endothelial dysfunction, resulting in compromised cerebral blood flow. Moreover, early evidence indicates that COVID-19-induced endothelial dysfunction [226] and NVC impairment can persist for several months following infection [21, 24]. This persistence raises concerns about the role of these vascular abnormalities in the neurological symptoms and cognitive deficits observed in individuals with long COVID [227, 228]. Disrupted NVC may lead to inadequate blood flow adjustments in response to neuronal activity, potentially affecting cognitive function, memory, and overall brain health [211].

Mitochondrial health is crucial for maintaining proper endothelial function and neurovascular coupling responses, as mitochondria play a vital role in energy production, regulation of oxidative stress, and cellular signaling [150, 152, 154, 155, 180, 229]. Healthy mitochondria ensure that endothelial cells can effectively manage vascular tone, blood flow, and the integrity of the BBB [152, 155, 229, 230]. They are also integral to the process of angiogenesis and the repair of damaged blood vessels. Emerging evidence suggests that post-COVID-19 syndrome is associated with mitochondrial dysfunction, which can lead to impaired energy metabolism, increased oxidative stress, and inflammation [231]. This mitochondrial impairment may contribute to cerebromicrovascular dysfunction observed in long COVID, potentially exacerbating neurocognitive symptoms and other neurological manifestations. Investigating the link between mitochondrial dysfunction and cerebromicrovascular health in post-COVID-19 patients is an intriguing area for future studies, which could uncover novel therapeutic targets to mitigate long COVID’s impact on brain health. There is also initial evidence that long COVID syndrome may also associate with capillary rarefaction [232] in the peripheral circulation. Further studies are needed to confirm that the cerebral circulation is also affected. Endothelial dysfunction, compounded by the effects of COVID-19, may contribute to a range of neurological manifestations in long COVID patients. Understanding the link between cerebromicrovascular dysfunction, endothelial impairment, and NVC disruption is crucial for unraveling the complex neurological consequences of conditions like long COVID. Further research is needed to fully comprehend the mechanisms underlying these vascular changes and their long-term impact on brain health.

### Cerebromicrovascular inflammation

SARS-CoV-2 infection can trigger cerebromicrovascular inflammation through direct and indirect mechanisms. Persisting microvascular inflammation can have far-reaching consequences on brain health and neurological manifestations of long-COVID syndrome, including BBB disruption, neurovascular dysfunction, and neuroinflammation.

SARS-CoV-2 can directly infect ACE2-expressing microvascular endothelial cells [38, 82, 83, 233–240]. This direct viral infection can trigger a local immune response, leading to inflammation within the cerebral microvessels. The heightened inflammatory status can disrupt the BBB, allowing the infiltration of immune cells and inflammatory mediators into the brain parenchyma. It is likely that SARS-CoV-2 may establish a persistent presence in tissues [241]. Accordingly, an increasing number of studies demonstrate persistent viral RNA and protein in various tissues, including the lungs, appendix, skin, breast tissue, and cerebrospinal fluid, months after the acute infection [241–247]. Persistent viral presence has been suggested as a potential mechanism underlying long COVID [241, 246, 247]. This persistence likely can lead to ongoing viral replication and low-level chronic inflammation in the vasculature, further contributing to cerebromicrovascular dysfunction in long COVID.

COVID-19 is associated with systemic inflammation, and the release of cytokines from peripheral organs likely exerts a significant impact on cerebral microvessels [248, 249]. Importantly, in long COVID, a persisting increase in inflammatory mediators has been observed, further contributing to cerebromicrovascular dysfunction and inflammation [250–254]. In addition to persistent viral RNA, recent studies have proposed that SARS-CoV-2 genetic material may integrate into the host genome [255, 256]. This integration could explain the prolonged presence of viral components and sustained immune activation observed in some long COVID patients.

The cerebral microvasculature has its local renin-angiotensin system (RAS), which plays a critical role in regulating blood flow and vascular tone [257–259]. SARS-CoV-2 infection may disrupt the balance of the local RAS, leading to vasoconstriction, inflammation, and oxidative stress within the cerebral microvessels. It is assumed that these alterations can exacerbate cerebromicrovascular inflammation and impair NVC, affecting neuronal function. Both COVID-19 infection and COVID-19 vaccines have been associated with perturbations in the systemic RAS, potentially leading to significant alterations in blood pressure regulation [260, 261]. It is conceivable that post-COVID hypertension may have secondary effects on the cerebral microcirculation, further complicating cerebromicrovascular health.

Recent evidence suggests that long COVID is associated with unique immune dysregulation, including a T cell signature characterized by enhanced CD4 + T cell responses and diminished CD8 + T cell activity. This imbalance likely contributes to chronic inflammation and persistent immune activation, exacerbating vascular injury and neuroinflammation [262].

Systemic COVID-19 (via increased CCL-11) and/or the presence of SARS-CoV-2 in the cerebral microvasculature can activate microglia, the resident immune cells of the brain [14, 15, 263–265]. Activated microglia release pro-inflammatory cytokines and chemokines, contributing to neuroinflammation and potentially exacerbating cerebromicrovascular dysfunction. In response to cerebromicrovascular inflammation and endothelial activation, activated leukocytes from the systemic circulation may infiltrate the brain. These immune cells can release additional inflammatory mediators and exacerbate neuroinflammation, further compromising brain health [266].

In response to SARS-CoV-2 infection, microvascular endothelial cells may undergo alterations in their secretory profile. This shift can result in an imbalance in the release of growth factors, cytokines, and matrix metalloproteinases (MMPs), potentially disrupting neuronal function and impeding the processes of neurogenesis [81–83]. Such changes in the microenvironment of the brain can have profound implications for overall neurological health.

Latent herpesvirus infections, such as those caused by Epstein-Barr virus (EBV) and cytomegalovirus (CMV), can persist in the vascular endothelium, where they remain dormant but can reactivate under certain conditions, such as immune stress [68]. Herpesvirus reactivation has been identified as a contributing factor to the pathogenesis of long COVID [67–69, 71, 267, 268]. Reactivation of latent herpesviruses can be triggered by the immune dysregulation and systemic inflammation induced by SARS-CoV-2 infection [68]. EBV and CMV reactivation can lead to a heightened inflammatory response, which likely exacerbates endothelial dysfunction, contributing to BBB disruption and neurovascular coupling impairment [62, 63, 269–276]. The interplay between SARS-CoV-2 infection and herpesvirus reactivation creates a complex inflammatory environment that may contribute to the ongoing neurological manifestations of long COVID [68]. Understanding how viral reactivation affects cerebromicrovascular function is essential for developing therapeutic strategies to mitigate the long-term cognitive and neurological consequences associated with long COVID.

The induction of autoantibodies following COVID-19 represents another potential mechanism contributing to the development of cerebromicrovascular inflammation. Several lines of evidence support the importance of autoantibodies in the immune pathogenesis of SARS-CoV-2 infection [277]. The development of autoimmunity is evidenced by extrafollicular B cell activation observed after acute COVID-19 infection, as anti-type I interferon autoantibodies.

Moreover, a significant overlap exists between symptom clusters observed in long COVID and those characteristic of systemic autoimmune disorders, such as systemic lupus erythematosus (SLE), antiphospholipid syndrome (APS), rheumatoid arthritis (RA), and undifferentiated connective tissue disease. This similarity suggests that SARS-CoV-2 infection may act as a trigger for the development or exacerbation of autoimmune responses. Several autoantibodies have been implicated in long COVID, either through direct pathogenic effects or associations with specific long-term manifestations, indicating that the virus can stimulate a broad-spectrum autoantibody repertoire. Notably, numerous autoantibody targets have been identified within the central nervous system, including intracellular and cell surface antigens. These targets are likely to contribute to the neurological symptoms frequently reported in long COVID patients by promoting neuroinflammation, disrupting neuronal integrity, or impairing blood–brain barrier function [277–279].

In summary, SARS-CoV-2 infection can promote cerebromicrovascular inflammation through various mechanisms, including direct viral invasion, persisting viral presence, altered local RAS function, herpesvirus reactivation, microglia activation, leukocyte infiltration, autoantibody production, and changes in endothelial secretion profiles. This heightened inflammatory status within the cerebral microcirculation has significant implications for brain health and can contribute to the neurological symptoms observed in conditions like long COVID. Understanding the multifaceted nature of cerebromicrovascular inflammation is essential for comprehending its role in the neurological consequences of SARS-CoV-2 infection.

### Small vessel disease: blood–brain barrier disruption and white matter hyperintensities

Cerebral small vessel disease (CSVD) is a prevalent cerebrovascular condition that plays a pivotal role in the development of cognitive impairment [25, 280]. It is characterized by pathological alterations in small perforating arteries, arterioles, venules, and capillaries within the brain’s intricate microvasculature. Recent evidence suggests that SARS-CoV-2 infection may contribute to the pathogenesis of CSVD, shedding light on its potential role in the cerebromicrovascular aspects of long COVID and its associated neurological consequences [25].

One of the hallmark features of CSVD is its propensity to disrupt the BBB, a vital protective boundary that regulates the passage of substances between the bloodstream and the brain [281]. In the context of CSVD, compromised BBB integrity allows for the infiltration of blood-borne substances, including inflammatory mediators, into the brain parenchyma. When considering COVID-19, both direct infection of endothelial cells within the cerebral microvasculature and the systemic inflammation induced by the viral infection are likely to exacerbate BBB disruption [12, 176, 282–287]. This, in turn, results in the leakage of pro-inflammatory factors into the brain microenvironment, contributing significantly to the neuroinflammation observed in long COVID [176].

In the context of long COVID, emerging research highlights the role of BBB disruption in the development of neurological symptoms, such as cognitive impairment often referred to as brain fog [176]. Recent findings indicate that BBB disruption not only is evident during acute COVID-19 infection but also persists in patients experiencing cognitive difficulties associated with long COVID [176]. Transcriptomic analysis of peripheral blood mononuclear cells suggests a heightened inflammatory status in these patients [176]. In vitro experiments further support these findings, demonstrating increased adhesion of peripheral blood mononuclear cells to human brain endothelial cells and elevated expression of inflammatory markers in brain endothelial cells exposed to serum from long COVID patients [176]. Collectively, these results underscore the significance of sustained systemic inflammation and persistent localized BBB dysfunction as key features contributing to the neurological manifestations observed in long COVID, particularly brain fog. [176].

Another characteristic manifestation of CSVD is the development of white matter hyperintensities (WMHs) observable on magnetic resonance imaging (MRI) scans [280, 288]. These WMHs represent regions of increased water content and altered tissue structure within the brain’s white matter, indicating areas of white matter damage. BBB disruption is thought to be a critical trigger for the genesis of WMHs [289–292]. Emerging evidence suggests a persistent increase in WMHs in the brains of older adults with a history of COVID-19, highlighting the potential long-lasting effects of cerebromicrovascular dysfunction in the context of long COVID [293–295]. These CSVD-associated WMHs can have profound implications for cognitive function and overall brain health.

To gain a comprehensive understanding of the neurological consequences of long COVID, it is imperative to delve into the intricate relationship between persisting systemic inflammation [176], presence of autoantibodies in the circulation [296–303], complement activation, cerebromicrovascular dysfunction, BBB disruption [176], and the development of WMHs. This exploration will shed light on the cerebrovascular aspects of long COVID and may pave the way for targeted interventions aimed at mitigating its neurological sequelae.

### Small vessel disease: cerebral microhemorrhages and ischemic lesions

Among the various manifestations of CSVD, two significant features are cerebral microhemorrhages (CMHs, also known as cerebral microbleeds) and ischemic lesions (including lacunar strokes), both of which likely contribute to the cerebromicrovascular aspects of long COVID [304–313].

CMHs are minuscule bleeding events that occur within the brain which can be best detected using T2*-weighted Gradient-Recall Echo (T2*-GRE) MRI sequences [169]. These CMHs serve as an important indicator of microvascular fragility and are considered a characteristic feature of CSVD. Epidemiological evidence has established a link between the presence of CMHs and cognitive dysfunction [169]. In recent studies, emerging evidence has suggested a potential association between COVID-19 and the development of CMHs [295, 314–331]. Investigating the relationship between SARS-CoV-2 infection and the occurrence of these microhemorrhages is of paramount importance in unraveling the cerebromicrovascular contributions to long COVID. Understanding how COVID-19 may influence CMHs is essential for comprehending the potential neurological consequences and cognitive impacts of long COVID.

Evidence of cerebral microemboli in COVID-19 patients has been documented, suggesting that microvascular occlusions could also contribute to cognitive and neurological symptoms associated with long COVID. These microemboli, potentially resulting from hypercoagulability and endothelial dysfunction, may lead to localized ischemic damage in the brain [332, 333]. Lacunar ischemic strokes, stemming from CSVD, constitute a significant portion, accounting for approximately 25%, of all ischemic strokes. These relatively small-scale strokes frequently give rise to not only VCID but also various neuropsychiatric and mood disorders, along with compromised mobility. COVID-19 has been associated with an increased risk of ischemic events, including lacunar strokes [295, 316, 317, 321, 322, 324, 325, 327, 334–344]. These ischemic lesions associated with COVID-19 potentially can lead to long-lasting cognitive impairment and other neurological symptoms, emphasizing the importance of exploring the interplay between cerebromicrovascular dysfunction and ischemia in the context of long COVID.

Both cerebral microhemorrhages and ischemic lesions have the potential to significantly impact brain health and contribute to the neurological manifestations observed in long COVID [314, 326, 327, 345]. Investigating the mechanisms underlying these cerebromicrovascular pathologies in the context of SARS-CoV-2 infection is essential for a comprehensive understanding of the neurological consequences of long COVID.

### Thrombotic sequelae

Emerging research has illuminated the propensity of SARS-CoV-2 infection to induce a prothrombotic state within the circulatory system, leading to a heightened risk of thrombotic events, including strokes and other cerebrovascular complications. Both arterial and venous thromboembolism are prevalent in patients with severe COVID-19. In patients with severe COVID-19 admitted to intensive care units, the occurrence of venous thromboembolic events varies, with estimates ranging from 20 to 35%. Deep venous thrombosis has been identified in a strikingly high percentage, affecting between 70 and 100% of patients who succumbed to COVID-19 [346–349]. Furthermore, arterial thrombosis, leading to conditions such as larger strokes, has been diagnosed in up to 4% of patients with COVID-19 hospitalized in intensive care units. COVID-19 is also associated with an increased risk of thrombotic events affecting the cerebromicrovascular system, leading to ischemic lacunar strokes. While hypercoagulability and thrombosis play crucial roles in acute COVID-19, emerging research suggests that thromboinflammation can persist in certain patients, even after viral clearance. Studies have documented the presence of viral spike proteins and RNA in recovered individuals months after their initial infection, potentially contributing to the ongoing thromboinflammatory processes and the formation of microclots [350–353]. It has been posited that microthrombi, especially in the cerebral circulation, play a role in contributing to long COVID [350–353].

The pathophysiology of thrombotic events in COVID-19 is multifaceted. The virus can directly infect endothelial cells, leading to endothelial dysfunction and injury [58, 354]. This endothelial injury can result in a hypercoagulable state characterized by increased clotting factor production and reduced fibrinolysis, promoting thrombus formation. Moreover, while SARS-CoV-2 infection of platelets is not productive, the virus can engage ACE2 and other potential receptors on platelets, leading to their activation and aggregation. Recent studies have implicated the envelope (E) protein of SARS-CoV-2 in coagulation disorders. In a cohort of COVID-19 patients and a murine model, hyperactivation of platelets was observed, driven by activation of the p38 MAPK-NF-κB signaling pathway via the CD36 transmembrane glycoprotein, stimulated by the E protein [355–357]. Elevations in D-dimer, von Willebrand factor (VWF), and factor VIII levels are commonly observed in patients with severe COVID-19. Additionally, the systemic inflammatory response induced by the virus can further enhance the prothrombotic environment. The presence of antiphospholipid antibodies has been identified as an additional prothrombotic factor in acute COVID-19 infection [358]. Lupus anticoagulant (LA) positivity has been reported in 50–90% of COVID-19 patients [359]. Additionally, the prevalence of IgG and IgM isotype anticardiolipin (aCL) antibodies and anti-β2 glycoprotein I (aB2GP1) antibodies was approximately 15%, while double antibody positivity (both aCL and aB2GP1) was observed in 25–50% of patients [360].

Thrombotic events within the cerebral microvasculature can have severe consequences for brain health. Ischemic strokes, resulting from the occlusion of small cerebral vessels by blood clots, can lead to persisting neurological deficits and cognitive impairments. Moreover, the potential formation of microthrombi within the microvasculature can disrupt blood flow and impair oxygen delivery to brain tissues. This disruption may exacerbate the neurological manifestations associated with long COVID-19 [350], even in cases where overt stroke is not visible on MRI and CT images. Understanding the thrombotic sequelae of SARS-CoV-2 infection is vital for elucidating the cerebromicrovascular contributions to long COVID and its neurological consequences.

### Exacerbation of amyloid pathologies and pathogenesis of Alzheimer’s disease

Alzheimer’s disease (AD) is a neurodegenerative disorder characterized by the accumulation of amyloid-beta (Aβ) plaques and tau protein tangles in the brain, leading to progressive cognitive decline.

Recent research has illuminated a potential connection between COVID-19 and the exacerbation of AD-related pathologies [361, 362], underscoring the critical role of the cerebromicrovascular system in both conditions.

Microvascular pathologies, including BBB disruption [363], endothelial dysfunction [364], NVC impairment, and cerebral amyloid angiopathy (CAA) [365], are recognized as early events in the pathogenesis of AD. A specific pathological subtype of inflammatory CAA, known as Aβ-associated angiitis (ABRA), is characterized by transmural granulomatous inflammatory vasculitis [366]. COVID-19, by impairing cerebromicrovascular health, is assumed to exacerbate the pathogenesis of neurodegenerative diseases. Additionally, the systemic inflammation induced by the virus, along with direct viral effects on both microvascular and brain cells, may lead to increased production, reduced clearance, and increased perivascular accumulation of Aβ. In certain cases, these changes may also promote inflammatory responses targeting vascular Aβ deposits, further accelerating neurodegenerative processes.

Importantly, individuals with mild cognitive impairment (MCI), a precursor to AD, may be particularly susceptible to the detrimental effects of COVID-19 on AD pathogenesis [21]. Research indicates that MCI patients who contract COVID-19 exhibit an elevated transition rate to dementia, emphasizing the significance of understanding the intricate relationship between these conditions [21]. Gaining a deeper understanding of how COVID-19 impairs microvascular health and exacerbates neurodegenerative diseases can inform potential therapeutic strategies to mitigate long-term neurological consequences and reduce the risk of AD development in affected individuals.

### Role of autoantibodies in COVID-related vascular pathologies

Autoantibodies have emerged as critical players in COVID-19-related cardiovascular pathologies [367–372] and may also contribute to the long-term vascular and neurological complications in long COVID [296–303, 373]. In the context of SARS-CoV-2 infection, the immune system can become dysregulated, leading to the production of autoantibodies target the body’s own cells, tissues, and organs [296–303]. An age-dependent increase in autoantibody levels has been observed in patients with severe COVID-19, suggesting that these autoantibodies may serve as markers for age-related stratification of the severe COVID-19 phenotype [374]. Evidence increasingly indicates that these autoantibodies may contribute to vascular injury by promoting endothelial dysfunction, inflammation, and thrombosis [367–369, 373]. Studies have identified anti-phospholipid antibodies, which are typically associated with autoimmune disorders like antiphospholipid syndrome, in patients with COVID-19 [375–378] These antibodies target phospholipids and phospholipid-binding proteins on endothelial cells, as demonstrated in anticardiolipin (aCL), anti-β2 glycoprotein I (aB2GP1), and lupus anticoagulant (LA) assays. Their activity can lead to endothelial cell activation, complement system activation, increased coagulation, and thrombosis. The presence of circulating antiphospholipid antibodies establishes a procoagulant state via multiple mechanisms, including inducing a proinflammatory and procoagulant endothelial phenotype, promoting leukocyte adhesion, cytokine secretion, and PGE2 synthesis, enhancing platelet aggregation, inhibiting anticoagulant pathways, disrupting fibrinolysis, and interfering with annexin A5.

The development of thrombosis typically requires a “second hit,” such as an additional proinflammatory or procoagulant trigger. COVID-19 infection may act as this secondary inflammatory trigger, exacerbating the risk of blood clot formation [379, 380]. In severe COVID-19 patients older than 50 years, autoantibodies targeting cardiolipin and platelet glycoproteins have been identified as the most significant markers for stratifying thrombotic risk [374]. Other autoantibodies that bind to endothelial cell surface proteins potentially exacerbate endothelial dysfunction by promoting vascular inflammation.

This autoantibody-driven endothelial activation has a cascading effect on microvascular integrity, impairing blood flow regulation, contributing to BBB disruption, and potentially facilitating neuroinflammation. One of the defining features of autoantibody-driven pathology in long COVID is the persistence of these antibodies well beyond the acute phase of infection. Even months after recovery, many patients continue to show elevated levels of autoantibodies, which may be a factor in the chronic vascular and neurocognitive symptoms of long COVID. Persistent autoantibody levels may sustain a state of low-grade inflammation and ongoing endothelial activation, particularly in the cerebral microvasculature. This chronic immune activation can prolong neuroinflammation, leading to further endothelial damage and BBB disruption, contributing to long-term neurological sequelae. Understanding the role of autoantibodies in COVID-related vascular pathologies opens potential therapeutic avenues. Immunomodulatory therapies may be beneficial in cases where autoantibodies are a prominent factor in vascular damage. Early identification of autoantibodies in COVID-19 patients, particularly those at high risk for long COVID, may enable tailored therapeutic strategies aimed at limiting vascular and neurocognitive complications.

In addition to the autoantibodies, there is evidence suggesting a role for anti-SARS-CoV-2 antibodies in the pathological manifestation of long COVID. In a cohort of blood donors experiencing long COVID, the adjusted analysis showed that a higher anti-nucleocapsid (NC) antibody was associated with a higher risk of long COVID, while higher ani-spike (S) antibody levels inversely correlated with manifestation of long COVID [381]. Although the precise molecular mechanisms remain unclear, it is plausible that molecular mimicry, formation of immune complexes, and bystander immune activation may all be implicated in the pathogenesis of long COVID.

### Potential side effects of COVID-19 vaccines

In the later stages of the pandemic, the majority of COVID-19 infections were observed in vaccinated individuals [382]. Consequently, many cases of long COVID were identified among vaccinated individuals [383]. This underscores the need for ongoing vigilance and management strategies for long COVID in both vaccinated and unvaccinated patients, as vaccinations primarily reduce severity but do not completely prevent chronic sequelae.

It is important to acknowledge that COVID-19 vaccines have played a pivotal role in curbing the virus’s spread and preventing severe illness. However, like any medical intervention, COVID-19 vaccines may elicit side effects. Understanding these side effects and their potential impact on cerebromicrovascular health is crucial [384], particularly in the context of long COVID [385]. Autoimmune/inflammatory syndrome induced by adjuvants (ASIA) is an immune-mediated condition characterized by autoimmune and inflammatory symptoms that arise in genetically predisposed individuals following exposure to environmental immunostimulatory factors. Among these factors, COVID-19 vaccination has been identified as a potential trigger capable of hyperstimulating the immune system and initiating autoimmune phenomena. The underlying mechanisms driving these processes include molecular mimicry, the production of immunoglobulins by age-associated B cells (ABC cells), and immunological responses stimulated by vaccine adjuvants [386]. One notable side effect associated with COVID-19 vaccines is the risk of vaccine-induced thromboembolism, which can lead to serious cerebrovascular events like strokes [385]. While such cases are rare, they have garnered significant attention due to their potential severity [387]. Vaccine-induced thrombosis primarily involves the formation of blood clots, often accompanied by low platelet levels, a condition referred to as vaccine-induced immune thrombotic thrombocytopenia (VITT). This immune-mediated process is characterized by platelet activation and the release of platelet factor 4 (PF4), activation of the coagulation cascade, development of microvascular damage and microbleeds, and the production of PF4 antibodies [388]. VITT has been identified as a rare but serious adverse event associated with adenoviral vector-based COVID-19 vaccines, including those developed by AstraZeneca and Johnson & Johnson. Neurological manifestations of ASIA include cognitive symptoms such as mental fogginess, memory deficits, absent-mindedness, anomic dysphasia, and inattention. More severe neurological symptoms, including stroke and multiple sclerosis-like presentations, may also occur. Additionally, ASIA-induced dysautonomia, stemming from the production of autoantibodies targeting receptors of the autonomic nervous system, further highlights the potential neurovascular impact of immune dysregulation [386, 389]. Although the incidence of VITT is exceedingly low, the neurological manifestations of ASIA are not uncommon. This underscores the importance of monitoring cerebrovascular and neurological health in individuals receiving these vaccines, particularly those with pre-existing cerebrovascular conditions or other risk factors. Research into the cerebromicrovascular effects of COVID-19 vaccines is ongoing, and it is crucial to determine whether these vaccines have any impact on cerebrovascular and brain health in long COVID patients. The appearance of autoantibodies targeting various cell types has been documented post-vaccination [390–395], and further research is needed to understand their potential long-term effects.

## Clinical implications

Evaluating cerebromicrovascular health in long COVID patients requires the utilization of advanced imaging techniques to gain insights into the intricate vascular changes within the brain. Several imaging modalities have proven valuable in this context, allowing for the non-invasive assessment of cerebral microvasculature and other relevant parameters. Susceptibility-weighted imaging (SWI) and T2*-weighted imaging are valuable for detecting microhemorrhages within the brain. Resting-state functional MRI (fMRI) technique can be used to evaluate functional connectivity within the brain and can help identify alterations in neural networks associated with cerebromicrovascular dysfunction. Transcranial Doppler ultrasonography (TCD) can be used to assess cerebral hemodynamics and NVC responses. Optical coherence tomography (OCT) and dynamic vessel analysis (DVA) are non-invasive imaging techniques that can assess the retinal microvasculature and neurovascular coupling responses, offering insights into microvascular health in the central nervous system. Identifying reliable biomarkers for cerebromicrovascular dysfunction (e.g., novel exosome-based biomarkers) is also essential for early detection and monitoring of long COVID-related cerebromicrovascular issues.

Developing targeted treatments for cerebromicrovascular dysfunction in long COVID is critical. Potential interventions may include anti-inflammatory agents, endothelial protectants, and therapies aimed at addressing specific mechanisms contributing to vascular dysfunction (e.g., anticoagulants). Exercise has shown promise as a beneficial treatment for long COVID [396]. It also has the potential to enhance cerebrovascular health and alleviate inflammation, which are essential factors for recovery from long COVID.

Interestingly, cognitive impairments such as memory deficits, attention difficulties, and executive dysfunction, as well as headache and dizziness are now being observed as presenting symptoms of acute infection with the new SARS-CoV-2 Omicron and its subvariants [397]. These symptoms can be part of the acute phase of infection as well as long COVID, which imply a shift in the virus’s neurotropism or its impact on the central nervous system. Moreover, it raises concerns about a potential increase in the neurological burden of acute infections, further complicating the long COVID sequelae. Future cohort studies will undoubtedly shed light on features of variant-dependent long COVID and its impact on the neurovascular system.

## Future research directions

Ongoing research initiatives are essential for deepening our understanding of cerebromicrovascular causes in long COVID overall, as well as its specific features in an older organism. Tracking the progress of these studies and their findings will inform future clinical practices and therapeutic developments. Future studies should prioritize investigating dysregulation of CBF as a critical area of impairment in individuals with long COVID. Understanding how microvascular endothelial dysfunction may interact with other cerebromicrovascular pathologies and its potential role in cognitive dysfunction can provide valuable insights into the underlying mechanisms of long COVID-related cognitive decline. These investigations may pave the way for targeted interventions aimed at preserving or restoring CBF and, subsequently, cognitive function in affected individuals. Longitudinal studies are imperative to observe the evolution of cerebromicrovascular dysfunction in long COVID over time. These studies can help determine the persistence of vascular issues and inform strategies for long-term management and prevention of neurological complications.

Identifying potential therapeutic targets for cerebromicrovascular dysfunction is a key research objective. Investigating novel treatments, such as drugs targeting specific pathways or cellular mechanisms like immunosuppressants, immunomodulators, autoantibody depletion, antivirals for reactivated herpesviruses, therapeutic vaccination, antioxidants, and targeting CCL11 for neurocognitive function holds promise for improving outcomes in long COVID patients. As a part of the Researching COVID to Enhance Recovery (RECOVER) Initiative, the National Institutes of Health (NIH) has initiated clinical trials across multiple locations in the USA [398, 399]. These trials are designed to investigate potential treatments for long COVID, encompassing a variety of approaches, including medications, biologics, and other therapeutic interventions. There are other trials supported by industry or philanthropy, and we believe that small, pragmatic, and adaptive clinical trials, coupled with deep molecular phenotyping, will hold keys to unraveling the mysteries of long COVID. In that regard, it will be particularly critical to enroll sufficient numbers of older adults in such trials to elucidate both the pathogenesis and the efficacy of interventions in this extraordinarily vulnerable population.

Long COVID shares several pathophysiological and clinical similarities with post-infection syndromes observed after acute infections with viruses such as EBV, CMV, dengue virus, and Chikungunya virus, among other non-viral pathogens [92, 400–406]. Both long COVID and post-infectious conditions caused by the other viruses share characteristic symptoms manifested mainly by chronic fatigue, neurocognitive dysfunction, myalgias, and arthralgias. These symptoms are often debilitating and persist long after the resolution of the acute infection. Immune activation, chronic inflammation, and autoimmune responses have been proposed as common underlying mechanisms in these conditions. However, given the diversity of viral families and their distinct mechanisms of infection and replication, a key scientific question remains: Is the virus itself or a component of the immune response the primary trigger for post-infectious syndromes, or do both factors act synergistically to drive the development of chronic symptoms? Exploring this interplay could provide crucial insights into the pathogenesis of long COVID and similar post-infectious syndromes.
